# Relationship between Neural Alteration and Perineural Invasion in Pancreatic Cancer Patients with Hyperglycemia

**DOI:** 10.1371/journal.pone.0017385

**Published:** 2011-02-28

**Authors:** Junhui Li, Qingyong Ma, Han Liu, Kun Guo, Feng Li, Wei Li, Liang Han, Fengfei Wang, Erxi Wu

**Affiliations:** 1 Department of Hepatobiliary Surgery, First Affiliated Hospital of Medical College, Xi'an Jiaotong University, Xi'an, Shaanxi, China; 2 Department of General Surgery, Second Affiliated Hospital of Medical College, Xi'an Jiaotong University, Xi'an, Shaanxi, China; 3 Department of Pharmaceutical Sciences, North Dakota State University, Fargo, North Dakota, United States of America; Univesity of Texas Southwestern Medical Center at Dallas, United States of America

## Abstract

**Background:**

Patients with higher levels of fasting serum glucose have higher death rates from pancreatic cancer compared to patients with lower levels of fasting serum glucose. However, the reasons have not been studied. The goal of the current study was to examine the neural alterations in pancreatic cancer patients with hyperglycemia and to identify the relationship between the neural alterations and perineural invasion.

**Methodology/Principal Findings:**

The clinical and pathological features of 61 formalin-fixed pancreatic cancer specimens and 10 normal pancreases as controls were analyzed. Furthermore, the expression of Protein Gene Product 9.5 (PGP9.5), Myelin P0 protein (MPP), NGF, TrkA, and p75 were examined by immunohistochemistry. The median number of nerves, the median area of neural tissue, and the median nerve diameter per 10 mm^2^ were larger in the hyperglycemia group than those in the euglycemia group (*p* = 0.007, *p* = 0.009, and *p* = 0.004, respectively). The integrated optical density (IOD) of MPP staining was lower in the hyperglycemia group than those in the euglycemia group (*p* = 0.019), while the expression levels of NGF and p75 were higher in the hyperglycemia group than those in the euglycemia group (*p* = 0.002, and *p* = 0.026, respectively). The nerve bundle invasion of pancreatic cancer was more frequent in the hyperglycemia group than in the euglycemia group (*p* = 0.000).

**Conclusions/Significance:**

Nerve damage and regeneration occur simultaneously in the tumor microenvironment of pancreatic cancer patients with hyperglycemia; the simultaneous occurrence may aggravate the process of perineural invasion. The abnormal expression of NGF and p75 may also be involved in this process and subsequently lead to a lower rate of curative surgery.

## Introduction

Pancreatic cancer (PanCa) is a fatal disease with almost 100% mortality rate. Although surgery is effective in 20% of patients, the tendency for local recurrence in the retroperitoneum has not been fully understood [1]. One of the main causes of local recurrence of the tumor is perineural invasion (PNI) [2], which is frequently found in PanCa (as high as 90%–100%) [3], [4]. As a common but nonspecific feature of PanCa [5], PNI is an important prognostic factor in PanCa. Also, PNI increases as the cancer becomes undifferentiated (i.e. MIA PaCa-2 PanCa cells) in PanCa [6].

Because the pancreas is an organ with rich innervations originating from the superior mesenteric plexus and the celiac plexus, we need to pay attention to both the extrapancreatic perineural invasion (with infiltration into one or more extrapancreatic neural plexuses) and the intrapancreatic perineural invasion [7]. It has been suggested that the perineurium (the sheath of connective tissue enclosing a bundle of nerve fibers) blocks neural invasion of PanCa. However, perineurium damage has been identified at the end of the nerve, at the site where the nerve is invaded by the blood vessels around nerves, and at the site invaded by reticular fibers [8]. As a result, PanCa cells easily invade from the weakened areas of the perineurium to the perineural space. If systemic or local factors continue to erode the strength of the perineurium, the extent of perineural invasion may be high.

Furthermore, the simultaneous appearance of PanCa and diabetes mellitus (DM) has long been recognized. Long standing DM increases the likelihood for PanCa to a moderate extent [9]; in most cases, however, new onset DM is one of the first clinical signs of PanCa [10], [11]. DM is an independent predictor of mortality from cancer of the pancreas [12]. Approximately 80% of PanCa patients have glucose intolerance or frank diabetes [13], [14]. In Korea, both elevated fasting serum glucose levels and a diagnosis of diabetes are regarded as independent risk factors for several major cancers, including stomach cancer, liver cancer, lung cancer, and PanCa; and the risk of these conditions increase with an increased level of fasting serum glucose [15]. In addition, the Korean patients with the highest fasting serum glucose had higher death rates from all cancers combined such as cancers of esophagus, larynx, stomach, colon, liver, bile duct, pancreas, lung, prostate, kidney, bladder, and brain as well as leukemia compared to the patients with the lowest level of fasting serum glucose [15]. Among these cancer types, the association between the death rates and fasting serum glucose levels was strongest for PanCa in men and in women [15], [16]. Also, neuronal glucose uptake depends on the extracellular concentration of glucose. Hyperglycemia in diabetes can cause up to four-fold increases in neuronal glucose levels. If the persistent episodes of hyperglycemia exist, then intracellular glucose metabolism leads to neuronal damage [17]. Such damages can lead to several debilitating problems since neuropathy frequently results in clinically significant morbidities, such as pain, loss of sensation, foot ulcer, gangrene, and amputation [18], [19].

Even though PNI and hyperglycemia are common phenomena in PanCa, research has not determined if the two factors generate a synergistic effect to promote the progression of PanCa. Recent studies [2] have demonstrated that PNI may be involved in reciprocal signaling interactions between tumor cells and nerves, and that the invading tumor cells may respond to pre-invasive signals within the peripheral nerve milieu. To examine the effect of hyperglycemia on the nerve tissue in PanCa, we analyzed the functional and morphological changes of nerves in the tumors of PanCa patients with and without hyperglycemia, and then we examined the relationship between the changes and PNI. In order to investigate the morphological features of the nerves in the new tumor microenvironment, Protein Gene Product 9.5 (PGP9.5) and Myelin P0 protein (MPP) were labeled within the nerve fibers and the myelin sheaths, by using immunohistochemical methods. Therefore, a better understanding of the hyperglycemia-nerve relationship may shed new light on the mechanisms of PNI in PanCa.

## Methods

### Ethical Permission

The study protocol and consent forms conform to the Declaration of Helsinki and were approved by the Ethical Review Board (ERB) Committee (The First Affiliated Hospital of Medical College, Xi'an Jiaotong University, China) and informed consent was obtained from all subjects.

### Patients

From January 1999 to March 2008, 513 pancreatic tumors were subjected to clinical examination at the First Affiliated Hospital, Xi'an Jiaotong University, China. Among them, 420 cases were pancreatic ductal carcinoma excluding intraductal papillary mucinous tumor, acinar cell carcinoma, and islet cell carcinoma. Ultimately, sixty-one patients who received a radical curative pancreatic operation with a pathologic diagnosis were investigated in this study. The median patient age was 58 years, and 22 of the patients were female. None of the patients received neo-adjuvant therapy before their initial operation. Regional lymph node dissection was performed in all patients, and portal vein resections were performed in 17 patients. None of the 61 patients received an adjuvant treatment.

### Grouping and clinical parameters

The 420 pancreatic tumors were divided into two groups according to the fasting blood glucose levels (an average level of long term for the patients with the history of DM and an average level of three days continuous after hospitalization) of the patients: (1) euglycemia/normal and (2) hyperglycemia/high. As summarized in Table 1, four clinical parameters were evaluated in each group, including: (1) abdominal pain, (2) curative operation, (3) history of DM, and (4) radiating pain. Of the patients with a history of DM, the cases were divided into a controlled group and an uncontrolled group (the level of blood glucose was not controlled to normal) according to the fasting blood glucose levels. Three clinical parameters were evaluated in each group, including: (1) abdominal pain, (2) curative operation, and (3) radiating pain.

**Table 1:** Comparison of clinical parameters in the different groups of pancreatic cancer (%).

Parameters	Abdominal pain	Radiating pain	History of diabetes	Curative operation
Euglycemia group	75.28(201/267)	28.84(77/267)	3.75(10/267)	16.86(45/267)
Hyperglycemia group	74.51(114/153)	30.72(47/153)	24.18(37/153)*	10.46(16/153)
Controlled group	80.00(8/10)	30.00(3/10)		10.00(1/10)
Uncontrolled group	56.76(21/37) *	21.62(8/37)		2.70(1/37) *

Chi-square test was performed to compare the clinical feature.

*, Hyperglycemia group versus Euglycemia group and Uncontrolled group versus Controlled group: *P*<0.05, respectively.

### Quantitative analysis of neural tissue

An analysis of pancreatic nerves was carried out by two independent researchers with the aid of digital image analysis and the Image Pro-plus software (Media Cybernetics, Maryland, USA). A charge coupled device (CCD) video camera and PROSCAN electronics 5000 (Hama, Germany) connected to the Image Pro-plus version 6.0 software (Media Cybernetics) were used to measure the entire area of each tissue section, number of nerves, and the nerve area. From the recorded measurements, the average area of nerves per 10 mm^2^ tissue areas and the percentage of tissue invaded by nerves were calculated. The innervation area per nerve and number of nerves per 10 mm^2^ of tissue area were also calculated.

### Immunohistochemistry

Immunohistochemical stainings were performed on 3 µm sections of the formalin-fixed paraffin-embedded samples using a standard avidin-biotin complex peroxidase technique. After deparaffinization with xylene and rehydration with serial gradient ethanol, the antigen was retrieved by heating the slides in 10 mM of citrate buffer (pH 6.0) for 6 min in a microwave. The endogenous peroxidase was blocked with 0.3% hydrogen peroxide. The slides were subsequently incubated with a blocking protein (Dako, CA, USA) for 10 min and primary antibody was added overnight at 4°C followed by rinsing. The antibodies used were: monoclonal anti-Protein Gene Product 9.5 (PGP9.5, Zhongshan Goldenbridge Biotechnology Co., LTD, Beijing, China), polyclonal anti-Myelin P0 protein (MPP, Beijing Biosynthesis Biotechnology Co., LTD, Beijing, China), polyclonal anti-nerve growth factor (NGF) (Millipore, MA, USA), polyclonal anti-TrkA (Cell Signaling Technology, Inc., MA, USA) and polyclonal anti-p75 (Sigma, MO, USA). The secondary biotinylated goat anti-rabbit antibody or goat anti-mouse antibody (Dako) was then applied for 30 min, followed by 30 min of incubation with streptavidin peroxidase (Dako LSAB+ HRP kit). After rinsing, the slides were visualized by diaminobenzidine (DAB) chromogen solution (Dako) and counterstained with routine hematoxylin, followed by dehydration through graded ethanol and mounting of the slides. The control group was selected from ten normal pancreatic tissues from euglycemic patients with normal body mass indices and whose ages had been matched with the experimental groups. To ensure the specificity of the primary antibodies, consecutive tissue sections were incubated in the absence of the primary antibody. No immunostaining was detected in these sections, showing the specificity of the primary antibodies used in this study.

### Histological examination and criteria for PNI evaluation

The resected specimens were fixed in 10% formalin at room temperature, and the size and gross appearance of the tumors were recorded. Each tumor was sectioned at intervals of 0.5 to 0.7 cm, and all of the sections were routinely processed and embedded in paraffin for histological examination. Serial sections (4 µm) of each tumor were processed. Then, the sections, stained with hematoxylin and eosin, were examined pathologically to confirm the diagnosis and assessed for tumor invasion of nerves. The protocol for evaluating the degree of PNI was modified from a previously described method [20]. The degree of PNI was classified into four grades as follows according to the new definition of PNI [21]: Ne0–no perineural invasion; Ne1–neurium invasion; Ne2–perineural space invasion; Ne3–nerve bundle invasion. Protocols for the study were approved by the ERB Committee for Human Research of Xi'an Jiaotong University.

Two investigators assessed the histological parameters and classified the degree of PNI. Whenever a discrepancy occurred, both investigators re-examined the slides to reach a consensus.

### Statistical analysis

Statistical analysis was performed with the SPSS 17.0 statistical program. Data were given as means ± standard deviation (S.D.). For statistical analysis, the Student- Newman-Keuls (SNK) test, one-way analysis of variance, and Chi-square tests were used. A *p* value less than 0.05 was considered significant.

## Results

### The effect of blood glucose on clinical parameters

Of all 420 cases, 153 (36.43%) had hyperglycemia, including new-onset diabetes and uncontrolled diabetes. The rest (267 cases) in the euglycemia group included no diabetic or controlled diabetic cases. As shown in Table 1, the hyperglycemia group had a lower frequency of abdominal pain, curative operation, and higher frequency of radiating pain. However, these differences were not statistically significant. Among the 153 hyperglycemia cases, 37 had a history of diabetes, compared with 10 of 267 euglycemia cases (*p* = 0.000, Chi-square test), and these differences were statistically significant. According to the fasting blood glucose levels, the cases with the history of diabetes were divided into the controlled or uncontrolled groups. The frequencies of abdominal pain and curative operation were significant lower in the uncontrolled group than that in euglycemia group (*p* = 0.017 and *p* = 0.025, respectively, Chi-Square test). The degree of radiating pain was not significant different between the two groups (*p* = 0.359, Chi-Square test).

### Comparison of neural changes between euglycemia group and hyperglycemia group

**Figure 1:**
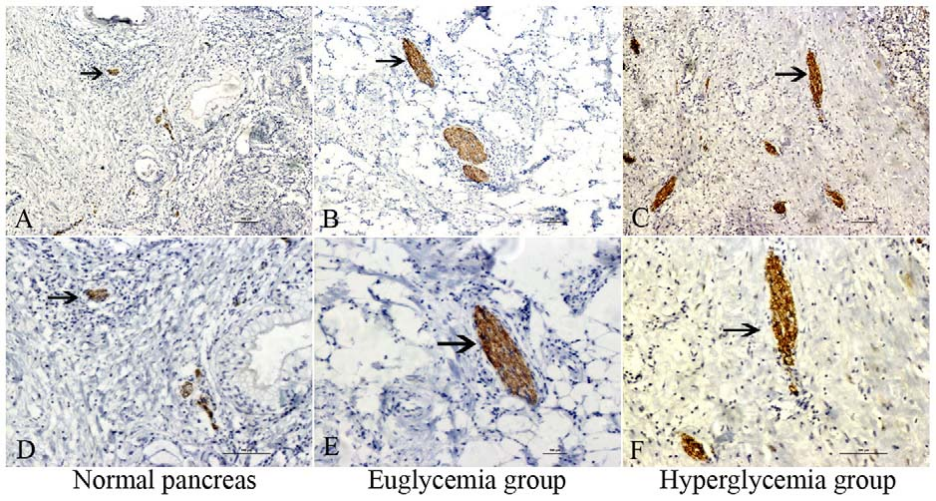
Nerve tissue immunostaining in different group. PGP 9.5 immunostaining in the normal pancreas (A, D, arrowhead), pancreatic cancer in euglycemia group (B, E, arrowhead), and hyperglycemia group (C, F, arrowhead). A, B, C show an original magnification of 100×, and D, E, F an original magnification of 200×. In the hyperglycemia group, comparable increases in the number, area, and diameter of nerve tissues were present. Figure arrowheads indicate the immunostainings.

**Figure 2:**
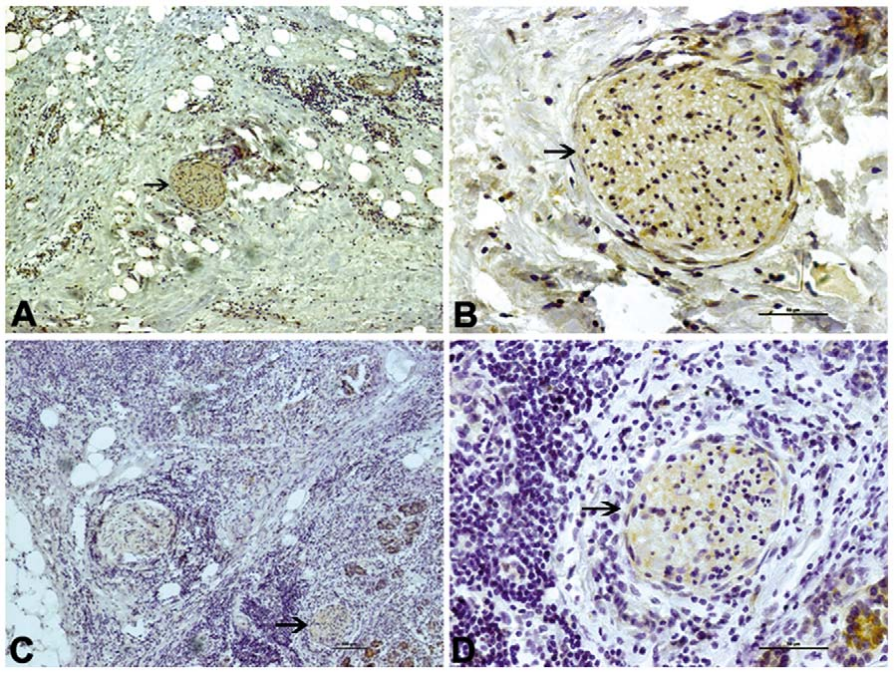
Myelin sheath immunostaining in different group. MPP immunostaining of pancreatic cancer tissue in the euglycemia group (A, B, arrowhead) and hyperglycemia group (C, D, arrowhead). The left panel shows an original magnification of 100×, and the right panel an original magnification of 400×. The frequency of moderate to strong MPP staining of nerves was significantly lower in the hyperglycemia group than in the euglycemia group. Figure arrowheads indicate the immunostainings.

The immunostaining of the neuron specific protein PGP9.5 was localized to the nerve fibers of pancreatic tissues (Fig. 1). The number of immunostainings represents the number and area of pancreatic nerve. The immunostaining of MPP which guides the wrapping process and ultimately compacts adjacent lamellae, was localized to the membrane of Schwann cells (Fig. 2). MPP expression in the nerves was significantly lower (*p* = 0.019) in the hyperglycemia group than in the euglycemia group, indicating dysfunction or demyelination of the myelin sheath. As shown in Table 2, the median area and median number of nerves per 10 mm^2^ of tissue area were significantly higher in the hyperglycemia group than in the euglycemia group (*p* = 0.007, *p* = 0.009, respectively). The median nerve area and median nerve diameter (*p* = 0.004) were significantly higher in the hyperglycemia group than in the euglycemia group.

**Table 2:** Comparison of nerve related parameters in the different groups.

Parameter	MTA(mm^2^)	MNN	MAN(µm^2^)	MND(µm)
Normal pancreas	86.94±4.85	1.93±0.13	1959.35±184.87	32.76±1.94
Euglycemia group	103.32±7.57	2.06±0.19	2002.38±126.34	33.19±2.95
Hyperglycemia group	124.64±5.38	2.24±0.25*	2279.98±191.69*	35.95±2.95*
Controlled group	109.23	2.21±0.32	2257.36±224.32	35.23±1.87
Uncontrolled group	125.34	2.31±0.38	2303.26±217.16	36.32±2.77

The Student- Newman-Keuls was performed to compare nerve related parameters in the different groups.

*, Hyperglycemia group versus Euglycemia group and Normal pancreas group. *P*<0.05, respectively.

MTA, Median tissue area analyzed (mm^2^); MNN, Median No of nerves per 10 mm^2^ tissue area; MAN, Median area of nerves per 10 mm^2^ tissue area (µm^2^); MND, Median nerve diameter (µm).

In contrast, MPP expression in the nerves was significantly lower in the uncontrolled group than in the controlled group. The median area and median number of nerves per 10 mm^2^ of tissue area were also higher in the uncontrolled group than all other groups. The median nerve area and median nerve diameter were higher in the uncontrolled group than all other groups.

### The cytokines/growth factors expression in different groups

**Figure 3:**
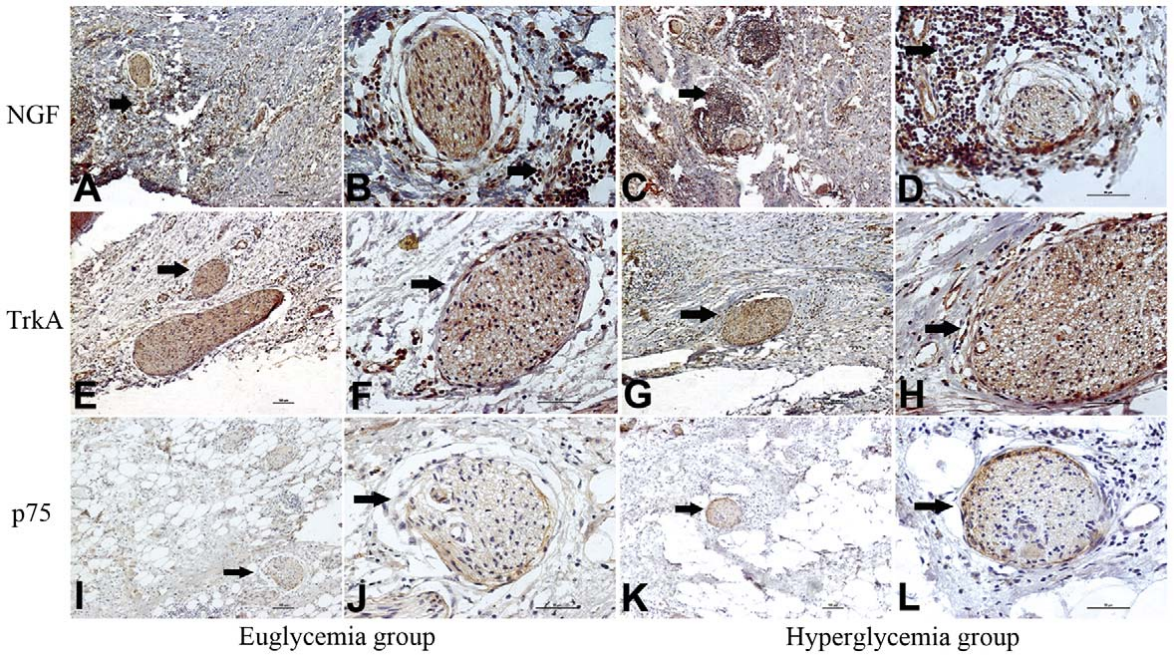
NGF, TrkA, and p75 immunostaining of pancreatic cancer tissue in the euglycemia group and hyperglycemia group. A, C, E, G, I, K show an original magnification of 100×, and B, D, F, H, J, L an original magnification of 400×. The frequency of moderate to strong NGF staining of cancer cells was significantly higher in the hyperglycemia group than that in the euglycemia group (arrowhead). The NGF stain of nerves was not significantly different between the two groups. The moderate to strong TrkA stainings were also present in the two groups (arrowhead) but with no significant difference. The frequency of moderate to strong p75 staining of nerves (arrowhead) was significantly higher in the hyperglycemia group than that in the euglycemia group. Figure arrowheads indicate the immunostainings.

**Figure 4:**
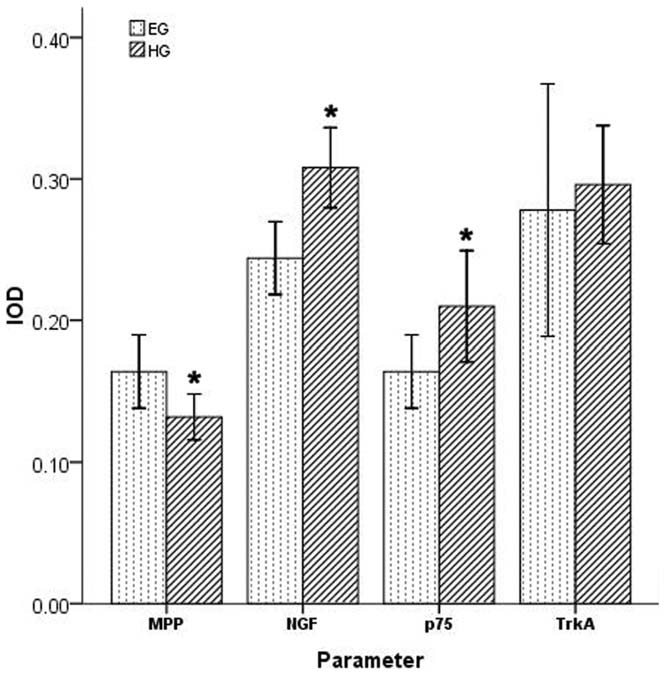
The comparation of four parameters in different group. Sixty-one patients who received a radical curative pancreatic operations at the First Affiliated Hospital, Xi'an Jiaotong University with a pathologic diagnose were investigated, which included 45 specimen in euglycemia group and 16 specimen in hyperglycemia group. The MPP, NGF, TrkA and p75 immunostainings were analyzed by Image-Pro Plus software. The integrated optical density (IOD) of MPP staining was lower in the hyperglycemia group than that in the euglycemia group (*p = *0.019). The IOD of NGF and p75 stainings were higher in the hyperglycemia group than that in the euglycemia group (*p = *0.002, *p = *0.026). The IOD of TrkA staining was not significantly different between the two groups.

The majority of cancer cells and nerve tissues showed distinct immunostainings of TrkA, P75NTR, and NGF localized to the cytoplasm. The nerve tissues had different expression levels of TrkA and p75NTR by immunostaining between the euglycemia group and hyperglycemia group. (Fig. 3). As shown in Fig. 4, the NGF staining in the cytoplasm of the cancer cells was significantly stronger (*p* = 0.002) in the hyperglycemia group than in the euglycemia group, indicating the NGF protein level of cancer cells was increased in the hyperglycemia group. The percentage of cancer area occupied by positive NGF staining cancer cells was significantly higher in the hyperglycemia group than in the euglycemia group. In the cross section of the nerves, the TrkA immunostaining of nerve fibers was equivalent in the two groups with no significant differences (*p* = 0.626). The p75NTR immunostaining had a peripheral pattern, where the surrounding area of nerve fibers was strongly stained and the central area was mildly stained in the hyperglycemia group; in contrast, the p75NTR immunostaining in the euglycemia group was light and uniform (*p* = 0.026).

### PNI evaluation

**Figure 5:**
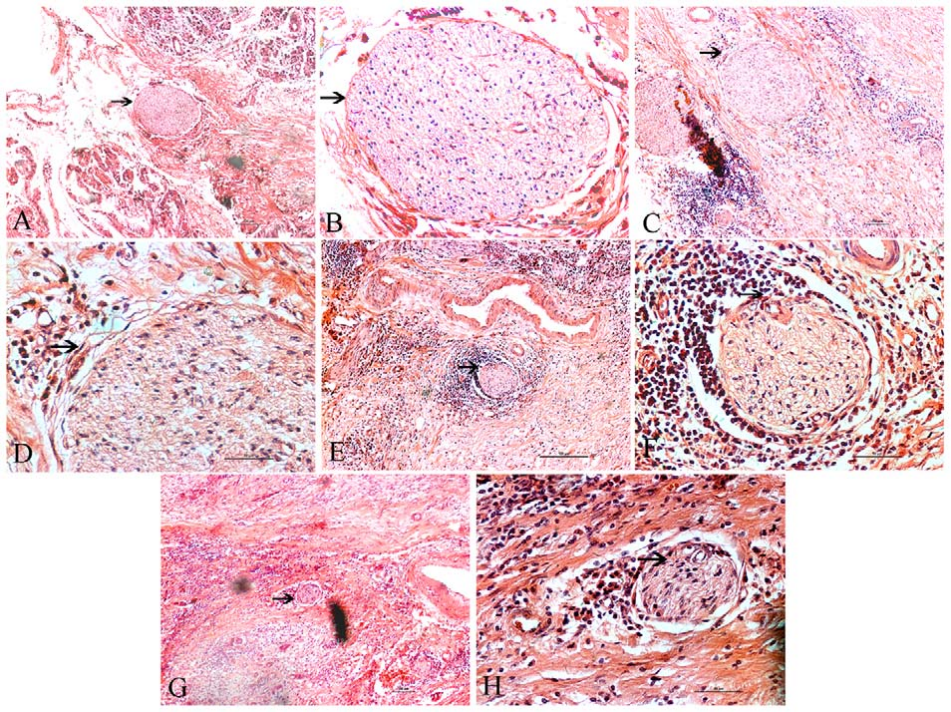
The classification of PNI in different group. Ne0–no perineural invasion; Ne1–neurium invasion; Ne2–perineural space invasion; Ne3–nerve bundle invasion. The nerve structures (A, B) and representative stages of the invaded neural structure for classification (C–H). (A) A nerve bundle surrounded by the continuous layered epithelioid sheets of the perineurium (arrowhead). (B) The perineural space, an internal space between the nerve bundle and perineurium (arrowhead). (C) Neurium invasion of cancer cells in the outer perineural space and attached to the perineurium (arrowhead). (D) Perineurial cells of perineurium between the nerve bundle and cancer cells (arrowhead). (E) Perineural space invasion of cancer cells in the perineural space (arrowhead). (F) Cancer cells invading the perineural space and compressing the nerve bundle smoothly. The border between cancer cells and nerve bundles do not contain perineurial cells or perineurium (arrowhead). (G) Nerve bundle invasion by cancer cells. Cancer cells irregularly compress the nerve bundle, clearly invading between nerve fibers (arrowhead). (H) Invasive cancer cells clearly seen between nerve fibers (arrowhead). A, C, E, G show an original magnification of 100×, and B, D, F, H an original magnification of 400×. Figure arrowheads indicate the nerves.

Representative images of normal structures of the nerves and the invading neural structure in various stages of classification in pancreatic tissues are shown in Fig. 5. A nerve bundle surrounded by the continuously layered epithelioid sheets is called the perineurium, while the internal space between the nerve bundle and perineurium is the perineural space. The degree of PNI was classified into four grades according to the presence of cancer cells in the different structures of the nerve tissues. As shown in Table 3, the total frequency of PNI was higher in the hyperglycemia group than that in the euglycemia group with no statistically significant difference. However, the frequency of the Ne3 invasion was significantly higher in the hyperglycemia group than that in the euglycemia group (*p* = 0.000, continuity correction).

**Table 3:** The classification of perineural invasion in the two groups.

Parameter	PNI (%)	Ne0	Ne1 (%)	Ne2 (%)	Ne3 (%)
Euglycemia group	84.44(38/45)	7	68.42(26/38)	21.05(8/38)	10.53(4/38)
Hyperglycemia group	93.75(15/16)	1	13.33(2/15)	20.00(3/15)	66.67(10/15)*
Controlled group	100(1/1)	0	0	0	100(1/1)
Uncontrolled group	100(1/1)	0	0	0	100(1/1)

One-way analysis of variance was performed to compare the classification of perineural invasion in the two groups.

*, Hyperglycemia group versus Euglycemia group: P<0.05.

## Discussion

In the current study, we present the neural alteration and extent of PNI in the primary tumors of PanCa patients in the context of glucose control. Our data suggest that hyperglycemia, a common confounding factor associated with PanCa, may contribute to PNI. We demonstrated that patients with hyperglycemia display reduced expression of MPP, and elevated expression of NGF and p75 in comparison to the patients with euglycemia. Furthermore, tumors from patients with hyperglycemia showed an elevation in the stage of PNI in comparison to tumors from the patients with euglycemia (Table 3).

Pain generation in PanCa seems to be multifactorial. It is speculated that abdominal pain in PanCa patients results from the invasion of cancer cells into neighboring organs [22]. Neuropathy seems to be caused by intra- and extra-pancreatic perineural invasion and damage to nerves by inflammatory and/or cancer cells [6], [23]–[25]. We did not find related studies about the correlation of PNI grade to pain. However, cancer cells can penetrate the perineurium and becomes intimately associated with Schwann cells and axons in the endoneurium. The neural remodeling shows how pancreatic neuropathic pain and visceral neuropathy are associated with altered pancreatic innervation in PanCa [26]. New data show that up to 80% of patients are either hyperglycemic or diabetic, both of which can be detected in the pre-symptomatic phase [14]. It is now recognized that impaired glucose tolerance, even without overt DM, may be a risk factor for the diabetic peripheral neuropathy (DPN). Neuropathic pain is a prominent early feature of DPN and can be severe despite minimal signs of DPN [27].

Accordingly, we reasoned that abdominal pain in PanCa patients with the history of DM should occur more frequently than those without the history of DM. In our study, the abdominal pain was more frequent, in the euglycemia group compared with the hyperglycemia group. The curative operation rate was significantly higher in the euglycemia group compared with the hyperglycemia group, indicating that the history of DM is associated with the lower curative operation rate in PanCa patients. With further investigation, we found that in those patients with a history of DM, the rates of abdominal pain and curative operation were significantly lower in the group that the level of blood glucose was uncontrolled to normal compared with the controlled group (Table 1). We believe that this phenomenon is not easily explained with a single theory because the microenvironment progressively worsens in these patients. The destructive effect of the cancer cells on nerves with compression is very mild, but the cancer can irritate the nerves and manifest as abdominal pain. When these patients were exposed in hyperglycemia state, hyperglycemia can exert a strong destructive effect on the fragile nerves, which promotes the progression from initial functional damage to late irreversible structural damage with or without attenuated pain.

The main morphological features such as a combination of demyelinization (damage to the myeline sheath of neurons), axonal degeneration of myelinated fibers, and degeneration with regeneration of unmyelinated fibers and endoneurial microangiopathy, with nerve fiber loss in its final stage, are present in the established neuropathy [19]. We found that the MPP expression level was lower in the hyperglycemia and blood glucose uncontrolled groups, which indicated the dysfunction or demyelinization of the affected nerve sheaths. In addition, we observed that the median number of nerves and median nerve diameters were greater in the hyperglycemia and blood glucose uncontrolled groups; this phenomenon suggests that nerve damage and repair are simultaneous in the new environment.

It is known that the observations of abnormal NGF levels in rodents and human diabetic nerves led to clinical trials to test the efficacy of NGF as a therapeutic agent for deficient neurotrophins. NGF treatment was shown to stimulate axon branching in the footpad, suggesting that NGF treatment exerted biological effects on cutaneous axons [28]. Our data demonstrated that NGF-positive cancer cells were evident and coincident with the improved nerves in the hyperglycemia group. We suggest that hyperglycemia may induce the NGF expression of cancer cells, which regenerates the affected nerves by stimulating axon branching and that NGF may be as potential key player in the generation of pancreatic neuropathy in PanCa [29]. Our observation of increased staining of p75NTR in the hyperglycemia group indicated dysfunction or demyelinization of the affected nerve sheaths and was consistent with a previous report suggesting that excessive NGF may cause apoptosis via p75NTR [30]. If nerve degeneration exceeds nerve regeneration in pathological conditions like DPN, nerve fiber loss may progress. However, if the specific therapy could be found to decrease nerve degeneration and/or increases nerve regeneration, pathological conditions, including nerve fiber degeneration and loss, could be improved [31].

In this study, we also found that the frequency and extent of PNI were increased in the hyperglycemia group. Thus, the special morphological features of affected nerves in the new microenvironment also may be correlated to the common phenomenon of PNI. First, the nerves increase in number, which offers more opportunities for cancer cells to invade and connect with the superior mesenteric plexus and the celiac plexus. The possibility of recurrence is increased even after curative operation. Second, the regenerated axons and demyelination can form a low resistance channel, which facilitates cancer cells to penetrate. When cancer cells penetrate the nerve and grow in the endoneurium, they affect the nerve processes and surrounding Schwann cells. Thus, all elements of the nerve are subject to damage and to be functionally altered. Furthermore, the cancer cells not only extend along the long axis of the nerve, but also tend to grow around the circumference, sometimes subsequently isolating the nerve from the surrounding tissue. In this manner, DM aggravates the PanCa prognosis by promoting PNI.

It should be noted that we have only analyzed the tumors from patients with curative operation in both euglycemia and hyperglycemia groups. We were not able to obtain suitable specimens from non-curable patients for two reasons. In some cases, the examination of the abdominal cavity during surgery revealed extensive metastasis, so we could not treat the cancer. In other cases, we could only perform palliative surgery such as drainage for jaundice because the tumor could not be resected. Despite these selections, our data showed that tumors from hyperglycemic patients exhibit an elevation in the stage of PNI in comparison to tumors from euglycemic patients (Table 3).

In summary, our results indicate that DM destroys pancreatic nerves with a pattern of pathological axons regeneration and demyelination in the tumor microenvironment, especially when blood glucose is not controlled. The consequence of destroyed nerves may aggravate a poor prognosis by two mechanisms: (1) destroyed nerves hide the abdominal pain of PanCa and hinder the early detection of PanCa and (2) destroyed nerves increase the frequency and extent of PNI in PanCa, which is a risk factor for local recurrence.
